# Supplementary material for: The causal relationship between the human gut microbiota and pyogenic arthritis: a Mendelian randomization study
Source: Front Cell Infect Microbiol. 2024 Nov 26;14:1452480. doi: 10.3389/fcimb.2024.1452480 (PMC11629706; doi:10.3389/fcimb.2024.1452480)
Supplement: Supplementary file 1 [file DataSheet1.zip › mendelian test/ebi-a-GCST90027581.csv_leave_one_out.pdf]

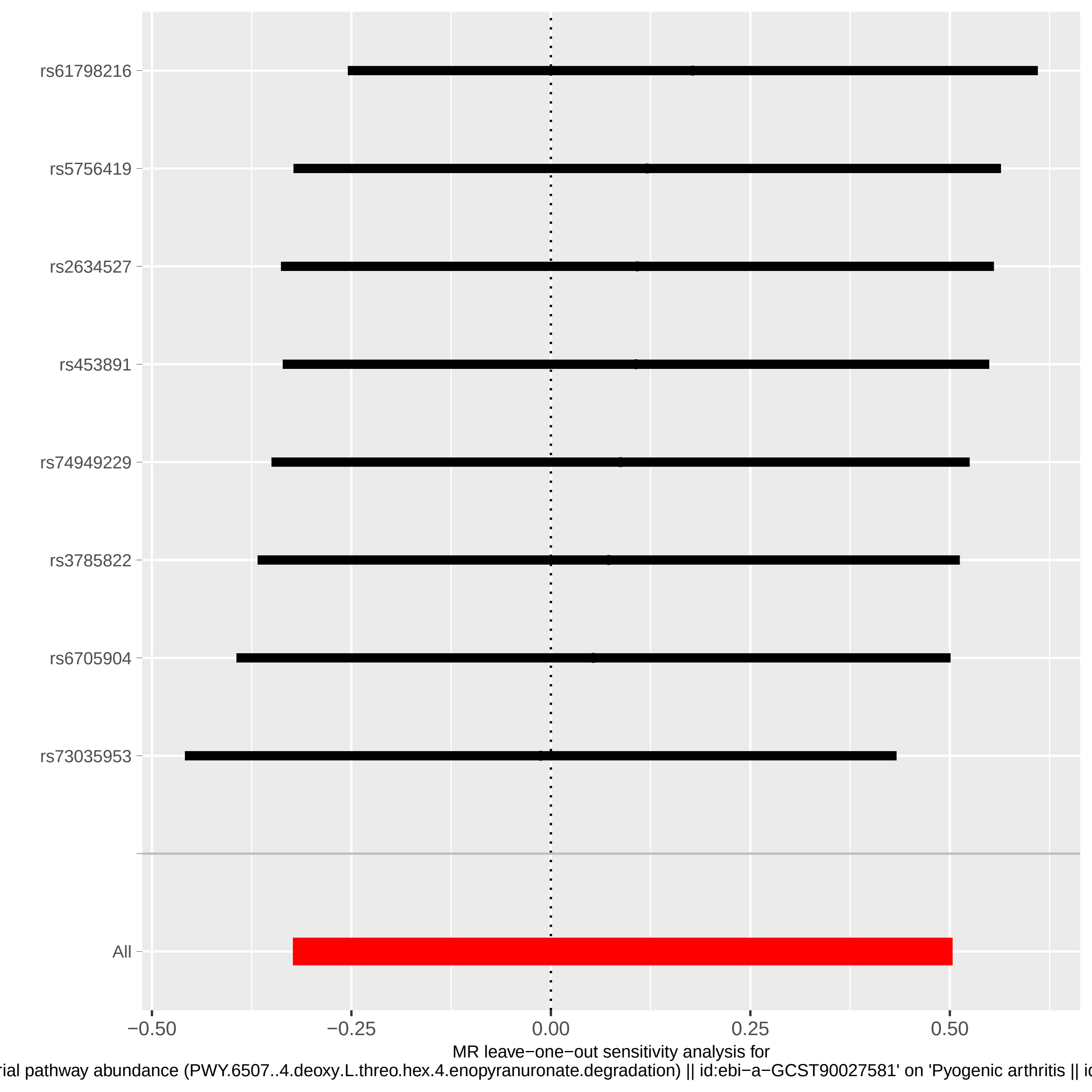
rs61798216
rs5756419
rs2634527
rs453891
rs74949229
rs3785822
rs6705904
rs73035953
All
−0.50
−0.25
0.00
0.25
0.50
MR leave−one−out sensitivity analysis for
rial pathway abundance (PWY.6507..4.deoxy.L.threo.hex.4.enopyranuronate.degradation) || id:ebi−a−GCST90027581' on 'Pyogenic arthritis || id
